# Associations between peripheral blood eosinophil counts in patients with systemic sclerosis and disease severity

**DOI:** 10.1186/s40064-016-3106-4

**Published:** 2016-08-23

**Authors:** Katsutoshi Ando, Tamao Nakashita, Norihiro Kaneko, Kazuhisa Takahashi, Shinji Motojima

**Affiliations:** 1 Division of Respiratory Medicine, Juntendo University Faculty of Medicine and Graduate School of Medicine, 2-1-1 Hongo, Bunkyo-Ku, Tokyo 113-8421 Japan; 2 Department of Rheumatology, Kameda Medical Center, 929 Higashi-Cho, Kamogawa-City, Chiba 296-8602 Japan; 3 Respiratory Internal Medicine, Kameda Medical Center, 929 Higashi-Cho, Kamogawa-City, Chiba 296-8602 Japan

**Keywords:** Eosinophil, Interstitial lung disease, Systemic sclerosis, Treatment

## Abstract

Increased levels of serum pro-fibrotic cytokines have been reported in patients with systemic sclerosis (SSc). Some of these cytokines also play an important role in the differentiation and migration of eosinophils. The aim of this study was to determine whether eosinophilic inflammation is caused in SSc. We retrospectively reviewed the peripheral blood eosinophil counts in 70 untreated patients with SSc and compared them with those in patients with other major collagen diseases. We additionally evaluated a possible association with disease severity. Eosinophil counts were significantly higher levels in patients with SSc than in those with other collagen diseases, whereas total leukocyte counts were not. Eosinophil counts correlated positively with both severe interstitial lung disease (ILD; r = 0.255, p = 0.033) and modified Rodnan total skin thickness score (m-Rodnan TSS) in SSc (r = 0.347, p = 0.003), but did not correlate with ILD severity in other collagen diseases. In conclusion, peripheral eosinophil counts were higher in patients with SSc than in those with other collagen diseases and were correlated with increased disease severity. Our data suggest that eosinophilic inflammation is involved in the pathogenesis and progression of SSc.

## Background

Systemic sclerosis (scleroderma, SSc) is an autoimmune connective tissue disorder characterized by microvascular injury, excessive fibrosis of the skin, and distinctive visceral changes involving the lungs, heart, kidneys, and gastrointestinal tract (Steen et al. 1994). Various clinical forms are recognized. These forms are generally classified into two major types on the basis of the extent of cutaneous fibrosis: (1) limited cutaneous SSc and (2) diffuse cutaneous SSc (LeRoy et al. 1988). Interstitial lung disease (ILD) and pulmonary hypertension (PH) are the most serious complications and common causes of premature death (Altman et al. 1991; Chang et al. 2003).

At present, several substances have been evaluated as biomarkers to assess the disease activity and its complications. Hasegawa et al. previously reported that serum levels of interleukin (IL)-4, IL-10, and IL-13 were elevated in patients with SSc (Hasegawa et al. 1997; Hasegawa 1998). In addition, the current study has reported that serum levels of IL-13 were increased in patients with SSc and the increase was positively correlated with the severity of the disease activity (Vettori et al. 2014). Other reports have suggested that serum levels of IL-33 were significantly higher in early SSc patients and those cytokines may play a critical role of promoting fibrosis in patients with SSc (O’Reilly 2013; Vettori et al. 2014).

Meanwhile, some of these cytokines play a key role in the differentiation and migration of eosinophils (Dubois and Brujinzeel 1994; Pope et al. 2001; Chen et al. 2004). In addition, levels of circulating eosinophil cationic protein (ECP) were increased in patients with SSc compared with those in healthy controls, and eosinophil activation is part of the inflammatory process in SSc (Gustafsson et al. 1991). In other words, such reports suggest that eosinophilic inflammation in patients might be caused in patients with SSc. However, controversy remains as to whether such inflammation might similarly be a symptom of in other collagen diseases and what the relationship is between that inflammation and the manifestations of disease in SSc. Accordingly, to determine whether the eosinophilic inflammation is related to the pathogenesis of SSc, we retrospectively assessed eosinophil counts in the peripheral blood of untreated SSc patients and compared these counts with those of individuals with other collagen diseases.

## Methods

### Study sample

For this retrospective review, we investigated the records of all patients that were diagnosed with SSc up to March 2013 in the Department of Rheumatology at Kameda Medical Center, a 1000 bed tertiary-care center. After screening, we identified 70 untreated SSc patients whose data were available for differential leukocyte counts, chest X-rays and computed tomography (CT). We excluded patients who lacked blood or imaging data, whose SSc treatment such as immunosuppressive agents had already been initiated, and who had comorbidities that potentially affected blood leukocyte and eosinophil counts such as infection, allergic or atopic diseases. SSc was diagnosed on the basis of American College of Rheumatology/European League Against Rheumatism (ACR/EULAR) 2013 criteria (Van den Hoogen et al. 2013) and the modified Rodnan total skin thickness score (m-Rodnan TSS) was assessed by two rheumatologists with twenty (TN) and forty (SM) years of experience in this field.

We compared eosinophil counts in 70 patients with SSc and in subjects with other major collagen diseases. Among these untreated patients, we identified 126 with rheumatoid arthritis (RA), 10 with polymyositis/dermatomyositis (PM/DM), 19 with primary Sjögren syndrome, 20 with systemic lupus erythematosus (SLE), and eight with mixed connective tissue disease (MCTD) according to a protocol similar to that used for SSc.

The presence of ILD was confirmed by two pulmonologists and one radiologist. The severity of ILD was visually assessed by chest CT images by the two pulmonologists independently and classified for its vertical extent by referring to a previously established method (Nakashita et al. 2014): grade 0, ILD not determined; grade 1, ILD extended across less than one-third of the lungs; grade 2, ILD extended across more than one-third but less than two-thirds of the lungs, and grade 3, ILD extended across more than two-thirds of the lungs. When there were disagreements regarding findings between two pulmonologists, an independent radiologist made the final determination of its severity. All clinical information was derived from medical records. This retrospective and observational study was approved by the ethics committee of our institution.

### Statistical analyses

We used the Chi squared test or Mann–Whitney test to compare the two groups of patients. Correlations of leukocyte and eosinophil counts with disease severity were evaluated by Spearman’s rank correlation analysis. To determine the factors correlated with eosinophil counts in patients with SSc, we used logistic regression multivariable analysis. These analyses were performed using SPSS Version 21. Data were expressed as means with standard deviations (SD). For all statistical analyses, a p value less than 0.05 was considered significant.

## Results

### Characteristics of patients

The clinical characteristics of the 70 patients with SSc are summarized in Table 1. The mean ages (±SD) at the onset of SSc-related symptoms, at diagnosis and at evaluation of eosinophil counts were 54.4 years (±12.1 years), 54.4 years (±11.8 years) and 59.3 years (±11.8 years), respectively. Of the patients, 87 % were women and 20 % had diffuse scleroderma. The mean modified Rodnan total skin thickness score (m-Rodnan TSS) was 6.2 (±4.8), and ILD was confirmed in 24 patients (34 %). Half of the patients with ILD had grade 2 (nine patients) or more severe ILD (grade 3, three patients), whereas ILD could not be detected by chest X-ray in seven patients with grade 1 ILD. Mean blood leukocyte and eosinophil counts were 5857 (±1430) and 230 (±145)/μL, respectively. Antinuclear, anti-topoisomerase I, and anti-centromere antibodies were positive in 92 % (49 of 55 patients), 13 % (7 of 54 patients), and 61 % (34 of 56 patients), respectively.

**Table 1:** Baseline characteristics of patients with SSc

	All patients (n = 70)	Eosinophil >7 % (n = 7)	Eosinophil <7 % (n = 63)	p value
Age at evaluation of eosinophils (years)	59.3 ± 11.8	60.9 ± 11.9	59.3 ± 12.0	p = 0.681
Age at onset of SSc symptoms (years)	54.4 ± 12.1	53.8 ± 12.1	54.5 ± 12.1	p = 0.846
Age at diagnosis	54.5 ± 11.8	54.5 ± 7.5	54.6 ± 12.5	p = 0.864
Male/female sex (n)	9/61	0/7	9/54	p = 0.583
Diffuse scleroderma-related disease [n (%)]	14 (20)	3 (43)	11 (17)	p = 0.137
Clinical findings				
m-Rodnan TSS	6.2 ± 4.8	11.1 ± 4.1	5.7 ± 7.0	p = 0.029
Skin edema [n (%)]	47 (67)	5 (71)	42 (67)	p = 1.0
Skin pruritus [n (%)]	12 (17)	4 (57)	8 (13)	p = 0.014
Pulmonary hypertension [n (%)]	1 (1)	0	1 (2)	p = 1.0
GI involvement [n (%)]	9 (13)	1 (14)	8 (13)	p = 1.0
Blood tests				
Total leukocyte counts (/μl)	5857 ± 1430	6100 ± 833	5830 ± 1516	p = 0.373
Eosinophil counts (/μl)	230 ± 145	552 ± 97	195 ± 97	p < 0.001
Positive for antinuclear antibody (>40) [n^a^ (%)]	49/55 (92)	5/5 (100)	44/50 (88)	p = 1.0
Positive for anti-topoisomerase I [n^a^ (%)]	7/54 (13)	1/5 (20)	6/49 (12)	p = 0.515
Positive for anti-centromere antibodies [n^a^ (%)]	34/56 (61)	3/5 (60)	31/51 (61)	p = 1.0
CT findings				
Presence of ILD [n (%)]	24 (34)	5 (71)	23 (37)	p = 0.107
ILD grade ≥2 [n (%)]	12 (17)	4 (57)	8 (13)	p = 0.014
X-ray findings				
Presence of ILD [n (%)]	17 (24)	5 (71)	12 (19)	p = 0.008
ILD grade ≥2 [n (%)]	12 (17)	4 (57)	8 (13)	p = 0.014

Plus minus data are presented as mean ± SD

*GI* gastrointestinal, *CT* computed tomography, *ILD* interstitial lung disease

^a^n/sample number

### Leukocyte and eosinophil counts in patients with SSc and other collagen diseases

**Fig. 1:**
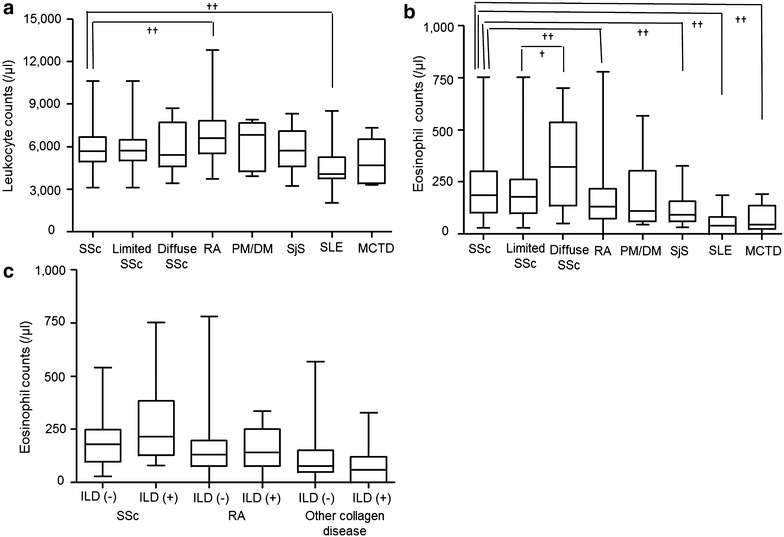
Peripheral blood leukocyte and eosinophil counts in SSc and other collagen diseases. Eosinophil counts were significantly higher in patients with SSc than those with RA, primary Sjögren syndrome (SjS), SLE or MCTD (**b**), but leukocyte counts did not follow that trend (**a**). Eosinophil counts were higher in patients with ILD than without ILD (275 vs. 201/μl, p = 0.092), but not in patients with RA or other collagen diseases (**c**) (^†^p < 0.05; ^††^p < 0.01)

In our retrospective review, leukocyte counts were lower in patients with SSc (5857 ± 1430/μL) than in those with RA (6801 ± 1723/μL, p < 0.001), but this difference was not observed when compared with patients with PM/DM (5324 ± 1644/μL, p = 0.365), primary Sjögren syndrome (5660 ± 1563/μL, p = 0.538), or MCTD (5542 ± 1989/μL, p = 0.491). In patients with SSc, there were no significant differences between limited and diffuse phenotypes (5807 ± 1391 vs. 6057 ± 1713/μL, p = 0.703) (Fig. 1a). However, eosinophil counts were higher in patients with SSc (230 ± 145/μL) than in those with RA (162 ± 125/μL, p = 0.004), primary Sjögren syndrome (119 ± 73/μL, p = 0.005), or MCTD (76 ± 66/μL, p = 0.002). Patients with diffuse SSc had the highest levels of these counts among our subjects (312 ± 200/μL). In patients with SLE, both leukocyte (4622 ± 1981/μL) and eosinophil counts (57 ± 69/μL) were lower than in those with other collagen diseases (Fig. 1b).

The comparison between patients with and without ILD is shown in Fig. 1c. Eosinophil counts were no different between patients with and without ILD in RA (144 ± 91 vs. 127 ± 115/μL, p = 0.944) and those with other collagen diseases (96 ± 84 vs. 107 ± 102/μL, p = 0.715). In SSc, those levels were higher in patients with ILD than in those without ILD (275 ± 171 vs. 201 ± 115/μL), but the difference was not statistically significant (p = 0.092). We then performed the same analysis between SSc patients with ILD grade ≥2 (n = 12) and <2 (n = 58). As a result, we noted that SSc patients with moderate (grade 2) or severe ILD (grade 3) had higher eosinophil counts than those with mild (grade 1) or no ILD (353 ± 204 vs. 205 ± 114/μL, p = 0. 018) (data not shown).

On the other hand, ILD was confirmed in 17 patients with SSc, 19 patients with RA and 17 patients with other collagen diseases by chest X-rays only, because mild ILD (grade 1) was difficult to be detected. We also performed the same analysis in the patients with and without ILD which was detected by chest X-rays, then eosinophil counts were higher in patients with ILD than in those without ILD (311 ± 196 vs. 205 ± 113/μL), but the statistical analysis also showed no statistically significant difference (p = 0.053). As for the patients with RA and with other collagen diseases, those were not different between patients with and without ILD.

### Correlation with the severity of disease

**Fig. 2:**
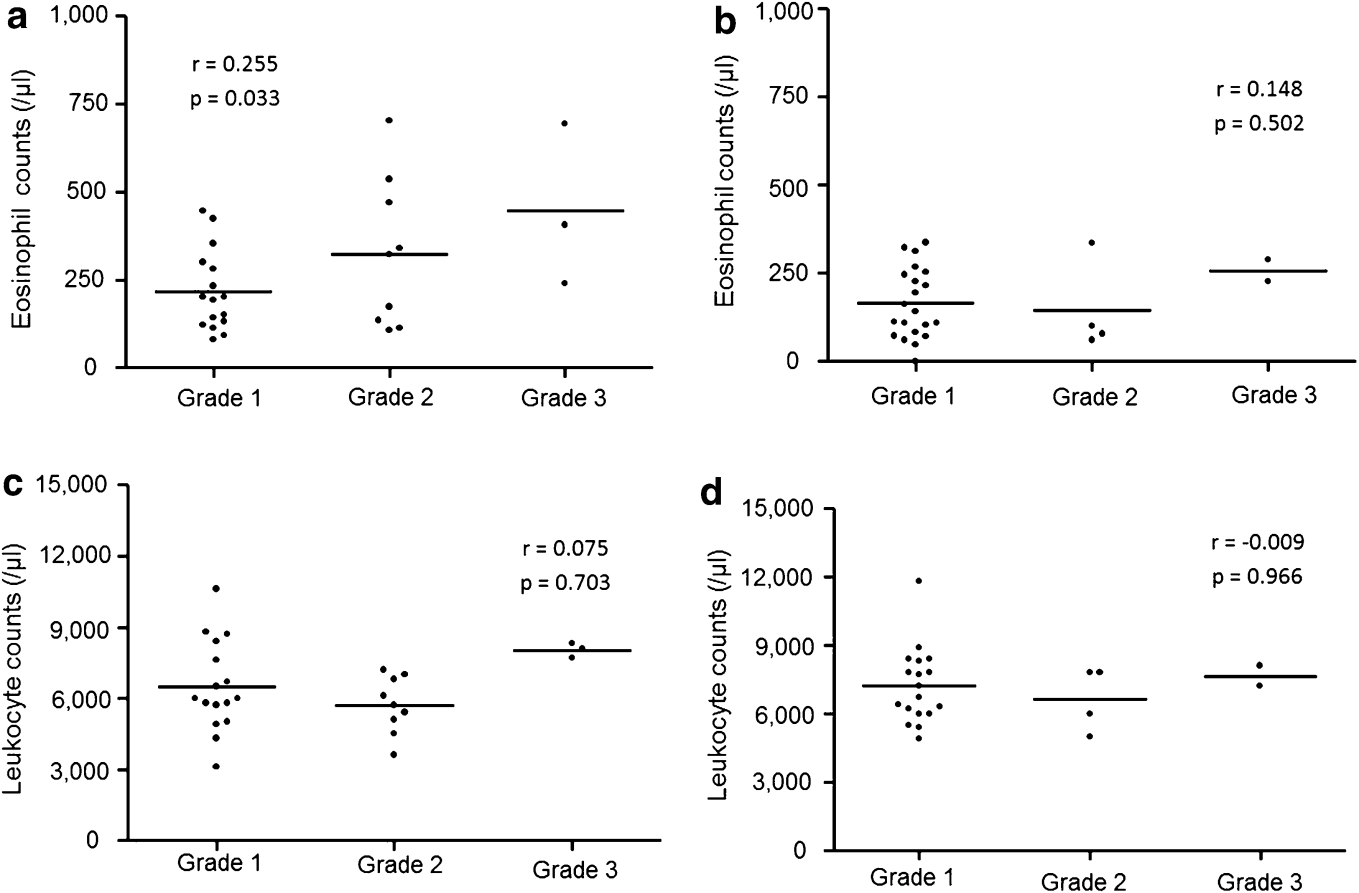
Correlations of eosinophil and leukocyte counts with ILD severity in patients with SSc (**a**, **c**) and RA (**b**, **d**). Eosinophil counts positively correlated with ILD severity in SSc (**a**), but not in RA (**b**). Leukocyte counts did not correlate to a statistically significant extent in both SSc (**c**) and RA (**d**)

The correlation of ILD severity with eosinophil or leukocyte counts in patients with SSc and RA is shown in Fig. 2. In SSc, ILD severity had a statistically significant positive correlation with eosinophil counts (r = 0.255, p = 0.033), but not with leukocyte counts (r = 0.075, p = 0.703). Meanwhile, in RA, neither eosinophil (r = 0.148, p = 0.502) nor leukocyte counts (r = −0.009, p = 0.966) were significantly correlated with ILD severity.

**Fig. 3:**
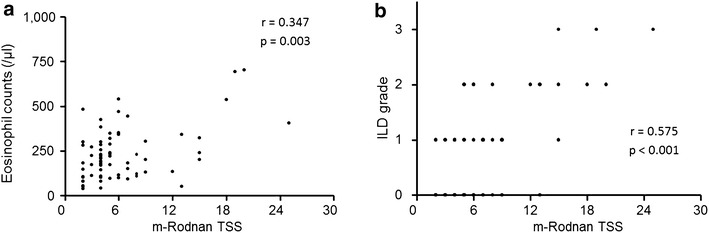
Correlation of m-Rodnan TSS with eosinophil counts (**a**) and ILD severity (**b**). m-Rodnan TSS correlated positively with eosinophil counts (r = 0.347, p = 0.003) and ILD grade (r = 0.575, p < 0.001)

When we next examined m-Rodnan TSS relative to eosinophil counts and ILD grade in patients with SSc (Fig. 3). The m-Rodnan TSS showed similar positive correlations to eosinophil counts and ILD grade (r = 0.347, p = 0.003 and r = 0.575, p < 0.001). Clearly, patients with SSc had the highest eosinophil counts among all the subjects considered in the present study, and those high counts correlated with the most severe categories of disease.

### Comparison of SSc patients with above and within normal limits of eosinophil counts

To identify the clinical characteristics of SSc patients with high eosinophil counts, we grouped these subjects according to the percentage of eosinophils in leukocytes. Although the number of peripheral blood eosinophils in healthy people has been reported to be 15–650/μL, the normal range of percentage of peripheral blood eosinophil has not yet been described (Krause and Boggs 1987). We then classified SSc patients into those with counts above 7 % (n = 7) and within normal limits (n = 63) as a cut-off point, referring to the report by Schulte et al. (2002), and compared their backgrounds (Table 1).

SSc patients with high eosinophil levels had higher m-Rodnan TSS values (11.1 vs. 5.7, p = 0.029) and proportions of patients with skin pruritus (57 vs. 13 %, p = 0.014), and this group included a larger proportion of patients with moderate or severe ILD (57 vs. 13 %, p = 0.014) than those within normal limits. A larger proportion of patients with the diffuse phenotype manifested high eosinophil counts than those within the normal range, but not to a statistically significant extent (43 vs. 17 %, p = 0.137). Age at onset of SSc symptoms, at diagnosis or at evaluation of eosinophil counts was not different between the two groups.

In the multivariate analysis, m-Rodnan TSS was determined as the factor that was independently associated with high eosinophil counts (hazard ratio [HR] 1.83; 95 % confidence interval [CI] 1.047–3.194, p = 0.034); however, the relationship between ILD grade and eosinophil counts (HR 0.074; 95 % CI 0–1925.12, p = 0.615) did not prove to be statistically significant.

## Discussion

The main findings of the present retrospective study were the following: (1) patients with SSc contained higher eosinophil counts than those with other major collagen diseases, and (2) eosinophil counts correlated positively with the severity of SSc. Accordingly, our data suggest that eosinophilic inflammation is involved in the pathogenesis and progression of SSc, and certain cytokines that cause eosinophilic inflammation are possible targets for treating SSc.

In SSc, cytokines such as IL-4, IL-6, IL-10, IL-13 and transforming growth factor-ß (TGF-ß) have been reported to play important pathogenic roles (Ludwicka et al. 1992; Needlemann et al. 1992; Patrick et al. 1995), and the serum levels of these cytokines have been shown to be higher in patients with SSc than in healthy volunteers (Needlemann et al. 1992; Hasegawa et al. 1997; Hasegawa 1998). On the other hand, Chiardola et al. investigated the prevalence of eosinophilia in RA, and reported that eosinophilia was not an indicator of the severity of RA (Chiardola et al. 2008). Some cases of primary sjögren syndrome have been reported to be complicated by eosinophilic inflammation; however, eosinophils have also been reported in biopsies of dermatomyositis lesions in 10–20 % of cases (Crowson et al. 2008; Waseda et al. 2015). However, the prevalence of eosinophils and the relationship with disease activity remain unclear. Our study similarly could not detect increased levels of eosinophils in those diseases.

Meanwhile, in patients with SSc-ILD in particular, macrophage-induced cytokines such as IL-6 and tumor necrosis factor-alpha were elevated (Hasegawa et al. 1997; Hasegawa 1998). Subsequently, Hasegawa et al. suggested that cytokines derived from type 2 T helper cells (Th2) and macrophages contributed to the pathogenesis of SSc and that macrophage-induced cytokines induced the development of ILD (Hasegawa et al. 1997; Hasegawa 1998). In addition, in previous clinical studies of SSc-ILD, the increased percentage of eosinophils in bronchoalveolar lavage fluid (BALF) was associated with decreased lung diffusing capacity for carbon monoxide and increased mortality (Bouros et al. 2002; Silver and Clements 2003). Hence, the inclusion criteria of patients in an important clinical trial yielded evidence of increased percentages of eosinophils in BALF (Tashkin et al. 2006), and an outgrowth of fibroblasts was observed in patients with an increased percentage of eosinophils in BALF (Scheja et al. 2007). In addition, in patients with bronchial asthma, we previously reported that the concentration of ECP and eosinophil-protein X (EPX) in sputum were higher than and significantly correlated with those in serum (Motojima et al. 1995; Koseki et al. 1996). These reports suggest that peripheral blood eosinophils counts can be affected by eosinophil accumulation in the lung, and thus denote that eosinophilic inflammation might be associated with the pathogenesis of SSc-ILD. However, we could not verify the participation of eosinophils in the progression of ILD. On this point, we considered that our assessment of ILD was qualitative, not quantitative, and consequently, a measurement bias might have led to our conclusion. Therefore, we will evaluate these points further through in vitro and prospective studies.

On the other hand, our data clearly determined that m-Rodnan TSS correlated with eosinophil counts as the independent factor, and was associated with elevated eosinophil counts. Gustafsson et al. examined skin biopsies by immunocytochemical techniques and reported that eosinophils were occasionally observed in samples of normal skin (from healthy control subjects). In addition, in patients with SSc, samples from unaffected areas revealed infiltration by eosinophils and extracellular deposits of ECP in the dermal layer, whereas the number of eosinophils was not increased in samples from areas of fibrotic skin (Gustafsson et al. 1991). In addition, eosinophils were reported to induce fibroblast proliferation, in contrast to neutrophils (Pincus et al. 1987; Gustafsson et al. 1991), and current investigations revealed that eosinophils promoted the expression of TGF-β, which plays a key role in fibrosis as well as inducing an epithelial to mesenchymal transition (Gharaee-Kermani and Phan 1998; Yasukawa et al. 2013). In SSc, fibroblasts derived from BALF had a great capacity to migrate and produce extracellular matrix proteoglycans, which was associated with eosinophil (Ludwicka et al. 1992). In other words, these previous reports suggest the possibility that eosinophil activation plays a role in the inflammatory process of SSc, and such inflammation induced the fibrosis. Accordingly, although detailed mechanisms of SSc and the impact of eosinophil on its pathogenesis remain unclear, we consider that this inflammatory process could be a target for delineating these mechanisms of SSc.

Our study had some limitations. First, as it was a retrospective analysis, we could not verify and evaluate some important data, such as pulmonary function, BALF, and pathological features of ILD. In addition, despite recent reports that KL-6 can be used as a lung fibrosis severity marker, and that the presence of elevated CXCL11 in BALF could serve as a prognostic factor for the decline of pulmonary function in SS-ILD (Sfriso et al. 2012; Kumánovics et al. 2014), we did not confirm those data nor determine serum pro-fibrotic cytokines such as IL-4 and IL-13. Consequently, in the current study, we identified a relationship of SSc pathogenesis with eosinophil counts only, but further evaluation of other biomarkers should be conducted. Second, because we included only untreated patients, the number evaluated here was relatively small, and we could not perform follow-up analyses. Therefore, we could not analyze in detail the effect of treatment on eosinophil counts. In other words, to elucidate detailed mechanisms of SSc and the role of eosinophils in the pathogenesis of ILD, our retrospective data should be re-evaluated prospectively. In addition, because the majority of SSc-ILD is characterized by a pattern of nonspecific interstitial pneumonia (King 2005), there is a discrepancy between the characterization of BALF and pathological appraisals. Therefore, to evaluate these mechanisms, we are currently planning in vitro and in vivo studies as well as prospective investigation.

## Conclusion

Our retrospective analysis revealed that levels of peripheral eosinophil counts were elevated in patients with SSc and were correlated with its severity. Considering previous reports, pro-fibrotic cytokines that cause eosinophilic inflammation might play an important role within the pathogenesis of SSc, and could be indicative of a treatment target.

## Abbreviations

BALF: bronchoalveolar lavage fluid
CI: confidence interval
CT: computed tomography
CXR: chest X-rays
IL: interleukin
ILD: interstitial lung disease
HR: hazard ratio
MCTD: mixed connective tissue disease
PM/DM: polymyositis/dermatomyositis
PH: pulmonary hypertension
RA: rheumatoid arthritis
SD: standard deviations
SDF: stromal-derived factor
SLE: systemic lupus erythematosus
SSc: systemic sclerosis
TGF-ß: transforming growth factor-ß
Th2: type 2 T helper cells
